# The Effect of School Bullying on Pupils’ Perceived Stress and Wellbeing During the Covid-19 Pandemic: A Longitudinal Study

**DOI:** 10.1007/s40653-022-00509-7

**Published:** 2022-12-22

**Authors:** Elizabeth J. Kirkham, C. F. Huggins, Fawns-Ritchie C

**Affiliations:** 1 Centre for Clinical Brain Sciences, University of Edinburgh, Edinburgh, UK; 2 Centre for Genomic and Experimental Medicine, Institute of Genetics and Cancer, University of Edinburgh, Edinburgh, UK; 3 Department of Psychology, University of Edinburgh, Edinburgh, UK; 4 Division of Psychology, University of Dundee, Dundee, UK; 5 Centre for Clinical Brain Sciences, Division of Psychiatry, Kennedy Tower, Morningside Terrace, EH10 5HF Edinburgh, UK

**Keywords:** Bullying, Covid-19, Adolescents, School, Lockdown, Perceived Stress, Wellbeing

## Abstract

Purpose: Establishing how the Covid-19 pandemic and related lockdowns have affected adolescent mental health is a key societal priority. Though numerous studies have examined this topic, few have focused on the wellbeing of pupils who experience school bullying. This is particularly important as pupils who experience bullying represent a vulnerable group at increased risk of mental illness. Therefore, we sought to investigate the relationship between experience of bullying and adolescent wellbeing during lockdown and subsequent re-opening of schools. Methods: We used the TeenCovidLife dataset to examine the relationship between experience of bullying and pupils’ perceived stress and wellbeing across three timepoints. Pupils aged 12–17 (n = 255) completed surveys during the first Covid-19 lockdown (May-July 2020), when they returned to school after the first lockdown (August-October 2020), and during the summer term of 2021 (May-June 2021). Results: Perceived stress was higher in the group of pupils that experienced bullying than in the group that did not, though this difference between groups was smaller during lockdown than when pupils were attending school in person. Pupils who were bullied showed lower wellbeing across all timepoints. For the full sample of pupils, wellbeing was lowest (and perceived stress highest) at Time 3, one year after the first Covid-19 lockdown. Conclusion:The findings challenge previous assumptions that Covid-19 lockdowns were associated with a generalised decline in adolescent mental health. Instead, the picture is more nuanced, with perceived stress, though not wellbeing, varying according pupils’ experiences of school bullying.

## Introduction

Mental health conditions can emerge following exposure to stress (Kendler & Gardner, 2016), and adolescence is a time of heightened risk for the development of such conditions (Kessler et al., 2007). Establishing ways in which the Covid-19 pandemic and related lockdowns have affected adolescent mental health is a key priority. Various studies and reviews examining this topic have reported reduced wellbeing and increased levels of stress among adolescents during the Covid-19 pandemic (Houghton et al., 2022; Jones et al., 2021; Meherali et al., 2021). While some of these studies have examined the relationship between feelings about the pandemic and mental ill health (e.g. Ellis et al., 2020; Oosterhoff et al., 2020), there have been limited attempts to distinguish the effects of the pandemic from the aforementioned increase in psychopathology that is already observed during adolescence. In addition, some studies have found increases in adolescent wellbeing during lockdowns and school closures (Penner et al., 2021; Widnall et al., 2020). Importantly, it is likely that no specific change in wellbeing has been universal, rather different people have responded in different ways (Houghton et al., 2022; Soneson et al., 2022).

Early evidence suggests that an individual’s experience of being bullied may be related to their mental health during Covid-19 lockdowns (Gomez-Leon, 2021; Soneson et al., 2022). Experiences of bullying in general are associated with reduced wellbeing (Arslan et al., 2021; Shaw et al., 2019) and higher rates of perceived stress (Canas et al., 2020; Estevez et al., 2009; Martinelli et al., 2011). Though bullying can take place in many locations, it is commonly reported within school settings (Karatzias et al., 2002). This raises the possibility that school closures during lockdown may have facilitated a reduction in the prevalence of bullying victimisation (Vaillancourt et al., 2021).

A small number of studies have examined whether the prevalence of bullying amongst young people changed following the beginning of the pandemic and associated lockdowns. These studies typically found either no change (Shin & Choi, 2021; Vaillancourt et al., 2021; Walters et al., 2021) or a reduction in bullying victimisation (Mastorci et al., 2021; Runkle et al., 2021; Soneson et al., 2022). Furthermore, emerging evidence suggests that expert concerns that cyber-bullying would increase during lockdown (Armitage, 2021; Englander, 2021) may not have come to pass. Studies examining cyber-bullying specifically have generally found no change in prevalence (Lessard & Puhl, 2021; Schunk et al., 2022), with one study finding a decrease in this form of victimisation (Vaillancourt et al., 2021). This suggests that school bullying did not simply move online when schools were closed. However, whilst evidence concerning the relationship between bullying prevalence and the Covid-19 pandemic continues to grow, it remains unclear what this means for adolescents’ wellbeing.

Therefore, we sought to investigate the relationship between experience of bullying and adolescent wellbeing during lockdown and subsequent re-opening of schools. To do this we used TeenCovidLife (Huggins et al., 2022), a longitudinal dataset examining the psychosocial and health impacts of Covid-19 on 12 to 17 year-olds living in Scotland, UK. Measures were collected at three timepoints corresponding to: the first Covid-19 lockdown (Time 1); pupils’ return to school after the first lockdown (Time 2); and the summer term of 2021 which coincided with pupils returning to school after another lockdown in early 2021 (Time 3). Specifically, we examined how pupils’ perceived stress and wellbeing scores changed over the course of the first pandemic period, and whether this differed between pupils who had experienced bullying and those who had not. We predicted that, overall, the group of pupils who experienced bullying would show higher perceived stress and lower wellbeing than the group of pupils who did not experience bullying. We further tentatively predicted that pupils who experienced bullying would show lower perceived stress and higher wellbeing during lockdown than when attending school, whilst pupils who did not experience bullying would show the opposite pattern. Due to the conflicting evidence from previous literature we did not make a directional prediction about how mental health would change for the sample as a whole.

## Methods

### Dataset

The data for this analysis were taken from TeenCovidLife, a longitudinal dataset examining the psychosocial and mental health impacts of Covid-19 on 12 to 17 year olds living in Scotland, UK. This dataset has been described in detail elsewhere (Huggins et al., 2022). Briefly, anyone living in Scotland who had access to the internet and was aged 12 to 17 when they joined TeenCovidLife could take part. Data were collected at three timepoints, using online surveys built using Qualtrics survey software (Qualtrics, 2020). Participants could complete the survey on a range of devices, including smartphone, tablet, and computer.

Multiple methods were used to recruit participants. Members of the Generation Scotland cohort (Smith et al., 2013) – a Scottish Health Study consisting of 24,000 adults – who had children aged 12–17 were contacted by email or letter inviting their children to take part. The Schools Health and Wellbeing Improvement Network (SHINE; https://shine.sphsu.gla.ac.uk/) is a network of over 500 schools in Scotland designed to facilitate school-based research by bringing together schools, researchers and policymakers. SHINE promoted TeenCovidLife to 138 high schools (Survey 1) and advertised TeenCovidLife via their monthly newsletters and social media (Survey 2 and 3). Both mainstream and social media were used to advertise TeenCovidLife to the general public and paid social media adverts were run on Twitter, Instagram and Facebook. No incentives were provided to participants for taking part in TeenCovidLife, except that all participants received a report of the results. Schools also received a report describing the results for pupils in their school.

The questions asked in each survey can be viewed in full at 10.5281/zenodo.5526056 (Huggins, 2021). The surveys were designed by Generation Scotland in collaboration with SHINE to assess general wellbeing and experiences during the pandemic, as well as to harmonise with other Generation Scotland and SHINE studies, including CovidLife (Fawns-Ritchie et al., 2021), and the Health Behaviour in School-Aged Children study (Inchley et al., 2020).

The first survey ran from 22nd May to 5th July 2020 (n = 5,543), which corresponded to the first school closure period when most school pupils in Scotland were doing school work at home; the second ran from 18th August to 14th October 2020 (n = 2,245), when most pupils in Scotland were returning to school for the first time since the initial lockdown; and the final survey ran from 12th May to 27th June 2021 (n = 597), one year after the first data collection period, when school pupils had returned to school after another school closure in early 2021. In total, 316 participants completed all three surveys.

### Participants

Complete relevant data across all three surveys was available for 255 participants. At 42.9%, the percentage of pupils reporting bullying was slightly higher than the 36% estimated by an 80-study meta-analysis of bullying rates in adolescents aged 12 to 18 (Modecki et al., 2014), and similar to Vaillancourt et al.’s (2021) more recent finding of 39.5% bullying victimisation towards the end of the first year of the pandemic (September – November 2020). Table 1 shows the number of participants by bullying status and sex. Participants were aged between 12 and 17 at baseline, with a mean age of 14.82 (SD = 1.46). As we only requested TeenCovidLife data pertaining to our planned analyses, no other demographic data are examined here.

**Table 1:** *Participant characteristics by experience of bullying*

Participant characteristic		Bullied n (%)	Not bullied n (%)
Sex	Male	29 (50.9%)	28 (49.1%)
	Female	83 (40.5%)	122 (59.5%)
Age group	12–14	57 (57.0%)	43 (43.0%)
	15–17	55 (34.0%)	107 (66.0%)
Total		112 (42.7%)	150 (57.3%)

### Measures

During the second and third TeenCovidLife surveys participants were asked “How often do other children or young people bully you in school?”, and could respond “all of the time”, “some of the time”, “never bullied” or “prefer not to say”. This question was adapted from a question asked to participants in the UK Millennium Cohort study (Connelly & Platt, 2014; “How often do other children bully you? Options: All of the time, some of the time, never”). We used these questions to create a measure of bullying. Bullying was coded as absent if participants chose “never bullied” at Time 2 and Time 3. Bullying was coded as present if a pupil indicated that they were bullied “all of the time” or “some of the time” on either survey 2 or 3. Where a participant responded “prefer not to say” to one survey, their experience of bullying was classified according to their response to the other survey. None of the participants responded “prefer not to say” to both surveys. Age in years, and sex assigned at birth (male/female/prefer not to say) were collected. Participants who responded “prefer not to say” were not included in the present analyses because there were so few. Due to the low variability in participant ages, pupils were divided into two age groups, “younger” (12–14 years) and “older” (15–17 years) adolescents.

All three TeenCovidLife surveys included commonly used measures of perceived stress and wellbeing. the Perceived Stress Scale 4 (PSS-4; Cohen et al., 1983) is a 4-item measure of stress experienced during the last month, and the WHO Wellbeing Index (WHO-5; WHO Regional Office for Europe, 1998), is a 5-item measure of wellbeing during the last two weeks. Total scores for these questionnaires were calculated by adding together the item scores. Possible scores on the PSS-4 ranged from 0 to 16, with higher scores indicating more perceived stress. For the WHO-5, a standardised percentage score was generated by multiplying this total score by four. As such, possible scores on the WHO-5 ranged from 0 to 100, with higher scores indicating higher wellbeing. Cases in which respondents had chosen “prefer not to say” for one or more item were excluded from analyses. Table 2 shows the mean scores for perceived stress and wellbeing at each timepoint, grouped by experience of bullying.

### Statistical Analyses

A mixed-model ANOVA was used to examine the effect of bullying on perceived stress across the three survey timepoints. Participant age at baseline and participant sex were included as control variables. Within-subject effects were examined using pairwise comparisons and interactions were examined using simple main effects. For each family of three tests a Bonferroni-adjusted alpha threshold of 0.017 was used to determine significance. The same process was then used to examine the effect of bullying on wellbeing across the three survey timepoints.

**Table 2:** *Wellbeing and perceived stress scores by bullying status*

Participant group		Perceived stress					Wellbeing		
	*n*	T1 Mean (*SD*)	T2 Mean (*SD*)	T3 Mean (*SD*)		*n*	T1 Mean (*SD*)	T2 Mean (*SD*)	T3 Mean (*SD*)
Experienced bullying	109	8.33 (3.11)	8.45 (3.39)	9.38 (3.35)		110	41.13 (20.70)	43.67 (20.82)	36.65 (20.28)
Did not experience bullying	145	7.52 (3.43)	7.13 (3.33)	7.94 (3.38)		145	43.81 (22.01)	48.03 (21.84)	41.30 (20.74)
Total	254	7.87 (3.31)	7.70 (3.41)	8.56 (3.44)		255	42.65 (21.45)	46.15 (21.47)	39.29 (20.63)

Note.SD = standard deviation. Perceived stress measured using the Perceived Stress Scale 4 (PSS-4; Cohen et al., 1983), wellbeing measured using the WHO Wellbeing Index (WHO-5; WHO Regional Office for Europe, 1998).

## Results

### Perceived Stress

**Figure. 1:**
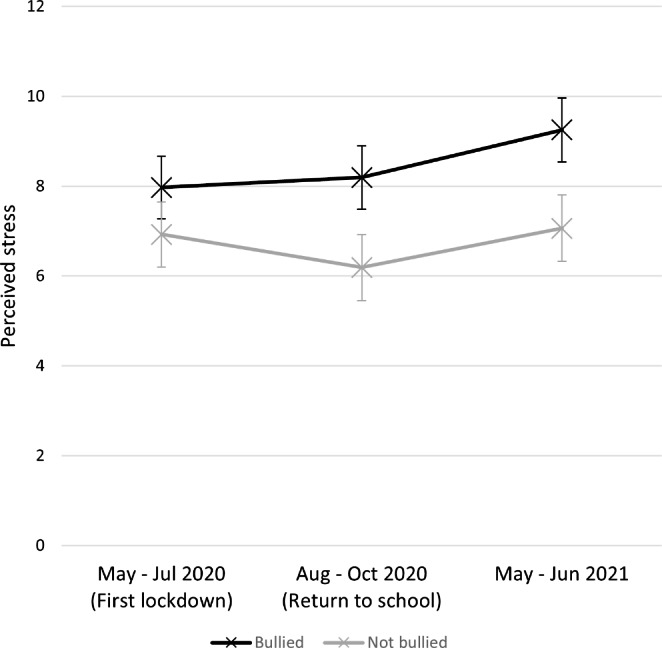
Perceived stress by bullying status over time. Note. Error bars represent 95% confidence intervals.

Perceived stress scores over time divided by bullying status are illustrated in Fig. 1. A mixed-model ANOVA was used to examine the effect of bullying on perceived stress across the three survey timepoints. There was a significant main effect of survey timepoint on perceived stress *F*(1.93, 473.52) = 9.79, *p* = < 0.001, partial *η*^*2*^ = 0.038. Pairwise comparisons using a Bonferroni-corrected alpha value of 0.017 indicated that, for the whole sample, perceived stress was significantly higher at Time 3 than at Time 1 (mean difference = 0.71, 95% CI, 0.12 to 1.30) and higher at Time 3 than at Time 2 (mean difference = 0.97, 95% CI, 0.42 to 1.52). There was no significant difference in perceived stress between Time 1 and Time 2.

There was a main effect of bullying on perceived stress *F*(1, 246) = 15.42, *p* < .001, partial *η*^*2*^ = 0.059, with participants who experienced bullying showing higher stress (*M* = 8.47) than participants who did not (*M* = 6.73). There was also a main effect of participant sex on perceived stress *F*(1, 246) = 12.76, *p* < .001, partial *η*^*2*^ = 0.0.049, with female participants (*M* = 8.39) showing higher stress than male participants (*M* = 6.80).

The interaction between bullying and survey timepoint had a significant effect on perceived stress (Fig. 1; *F*(1.93, 473.52) = 3.66, *p* = .028, partial *η*^*2*^ = 0.015). To interpret this interaction, simple main effects were examined using a Bonferroni-adjusted alpha level of 0.017. As before, participant sex and age group were included as control variables. There were significant simple main effects of bullying on perceived stress at survey timepoints 2 (*F*(1,246) = 15.05, *p* < .001) and 3 (*F*(1,246) = 17.62, *p* < .001), with participants who had been bullied showing significantly higher perceived stress scores than those who had not been bullied at timepoint 2 (mean difference = 2.00, *p* < .001, Bonferroni-adjusted 95% CI 0.99, 3.02) and timepoint 3 (mean difference = 2.19, *p* < .001, Bonferroni-adjusted 95% CI 1.16, 3.22). However, although participants who had been bullied still showed slightly higher mean stress scores than those who had not been bullied at timepoint 1, the difference between groups was smaller (mean difference = 1.05, *p* = .04, Bonferroni-adjusted 95% CI, 0.04 to 2.05), and did not reach significance using the Bonferroni-adjusted alpha level of *p* < .017 (*F*(1,246) = 4.21, *p* = .04).

Simple main effects were then used to examine the effect of time on perceived stress within each of the bullying groups. For the group who experienced bullying, the effect of time was significant (*F*(2,210) = 9.40, *p* < .001). Pairwise comparisons indicated that Time 3 involved significantly higher stress than either Time 1 (mean difference = 1.28, *p* = .001, Bonferroni-adjusted 95% CI, 0.44, 2.12) or Time 2 (mean difference = 1.06, *p* = .003, Bonferroni-adjusted 95% CI, 0.30, 1.82), but that there was no significant difference in stress between Time 1 and Time 2 (mean difference = -0.22, *p* = 1.00, Bonferroni-adjusted 95% CI, − 0.92, 0.48).

For the group who were not bullied, the effect of time was also significant (*F*(2,282) = 4.18, *p* = .016). Pairwise comparisons using Bonferroni-corrected 95% confidence intervals indicated that Time 2 was significantly less stressful than Time 1 (mean difference = -0.73, *p* = .04, Bonferroni-adjusted 95% CI, -1.45, − 0.02) or Time 3 (mean difference = -0.87, *p* = .029, Bonferroni-adjusted 95% CI, − 1.68, − 0.07), though neither of these comparisons met the Bonferroni-corrected alpha level of 0.017. There was no significant difference between Time 1 and Time 3 (mean difference = -0.14, *p* = 1.00, Bonferroni-adjusted 95% CI, − 0.98, 0.70).

This suggests that for the pupils who experienced bullying, perceived stress was similar during lockdown (Time 1) and upon their return to school (Time 2), before rising to its highest level one year after the first lockdown (Time 3). Pupils who did not experience bullying showed a different pattern, such that they had similar levels of stress during lockdown (Time 1) and one year later (Time 3), but lower levels of stress around the time they returned to school following the first lockdown (Time 2).

### Wellbeing

**Figure. 2:**
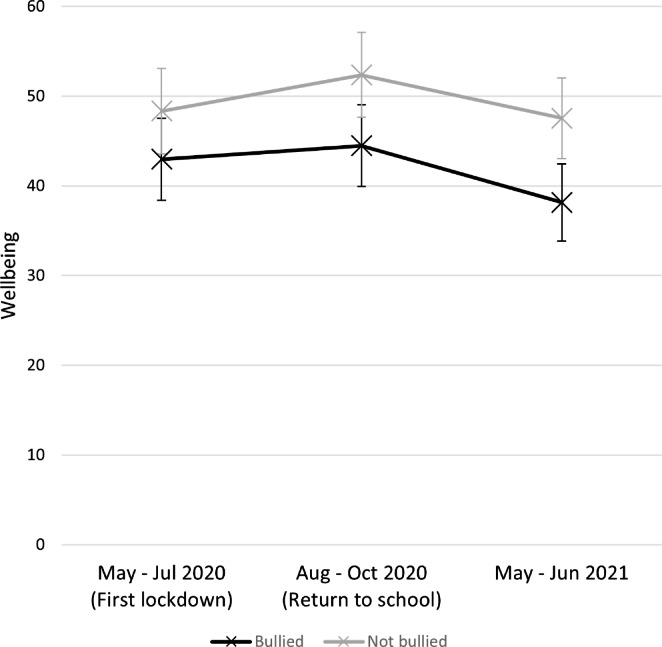
Wellbeing by bullying status over time. Note. Error bars represent 95% confidence intervals.

Wellbeing scores over time divided by bullying status are illustrated in Fig. 2. A mixed-model ANOVA was used to examine the effect of bullying on wellbeing across the three survey timepoints. There was a significant main effect of survey timepoint on wellbeing *F*(2, 494) = 6.61, *p* = .001, partial η^2^ = 0.026. Pairwise comparisons indicated that, for the full sample, wellbeing was significantly higher at survey timepoint 2 than at survey timepoint 3 (mean difference = 5.58, 95% CI, 1.80 to 9.35). However, there was no significant difference in wellbeing between timepoint 1 and either of the other timepoints. There was a main effect of bullying on wellbeing *F*(1, 247) = 7.45, *p* = .007, partial η^2^ = 0.029, with participants who experienced bullying showing lower wellbeing (*M* = 41.86) than participants who did not (*M* = 49.41). There was also a main effect of participant sex on wellbeing *F*(1, 247) = 11.34, *p* = .001, partial *η*^*2*^ = 0.044, with male participants (*M* = 50.29) showing higher wellbeing than female participants (*M* = 40.98). No further main effects reached significance, and the interaction between bullying and survey timepoint was not significant (*F*(2,494) = 0.87, *p* = .42, partial *η*^*2*^ = 0.004).

## Discussion

This research examined the relationship between experience of bullying and Scottish school pupils’ wellbeing and perceived stress across three timepoints following the beginning of the Covid-19 pandemic. The timepoints covered the first Covid-19 lockdown, pupils’ return to school after the first lockdown, and the summer term of 2021. Pupils who had been bullied showed higher perceived stress and lower wellbeing than pupils who had not been bullied. However, during lockdown, levels of perceived stress in those who experienced bullying were closer to the levels of perceived stress in those who had not been bullied. Levels of perceived stress and wellbeing amongst the whole sample of participants varied across time, with pupils showing the highest levels of perceived stress and lowest levels of wellbeing during the summer term of 2021, approximately one year after the first Covid-19 lockdown.

As predicted, the group of participants who had experience of bullying had higher perceived stress scores and lower wellbeing scores when compared to the group of participants who did not experience bullying. These findings align with previous research highlighting the relationship between bullying and poorer mental health (Arslan et al., 2021; Canas et al., 2020; Estevez et al., 2009; Martinelli et al., 2011; Shaw et al., 2019). Importantly, the difference between the two groups on the measure of perceived stress was smaller when measured during lockdown than when the pupils were attending school. Furthermore, whilst pupils who were not bullied showed an improvement in their stress levels when returning to school after the first lockdown, this pattern was not present amongst pupils who experienced bullying. Whilst we were unable to measure perceived stress prior to the Covid-19 lockdown, this finding aligns with other studies which suggest that lockdown may have been associated with positive changes in mental health for pupils who experience bullying (Gomez-Leon, 2021; Soneson et al., 2022).

Overall, our findings do not support the suggestion that lockdown was worse for pupils’ mental health than being in school; although the group of pupils who were not bullied saw a slight improvement in stress levels upon their initial return to school, these stress levels had risen again by the time they were measured the following year (whilst they were in school). Our results are in line with Houghton et al. (2022) who found that, relative to pre-Covid levels, pupils’ depression scores were higher both during lockdown and when pupils had returned to school. Furthermore, when compared to pre-Covid data, internalizing symptoms and reported isolation were significantly higher after schools had re-opened, but were not significantly higher during lockdown (Houghton et al., 2022). Whilst the present analysis does not include pre-lockdown measures, the fact that, overall, perceived stress was lower during lockdown than approximately one year later suggests that lockdown itself cannot fully explain the widely reported deterioration in adolescent mental health during the Covid-19 pandemic.

One possible reason for heightened stress and lower wellbeing at Time 3 concerns the wider context in which the data were collected. Specifically, the data for Time 3 were gathered at a time when pupils in Scotland usually sit national examinations. However, in 2021, as a result of the Covid-19 pandemic, national exams were cancelled in Scotland. In these cases final grades were instead decided by teacher-estimated grades based on assessments and/or coursework. The uncertainly about these assessments may have led to increased rates of perceived stress. Indeed, concerns around assessment have been previously reported as a source of stress for pupils during the pandemic (Fisher et al., 2021; Soneson et al., 2022; Widnall et al., 2020).

The findings of our research should be interpreted in light of a number of limitations. Firstly, the sample is not representative of the Scottish adolescent population. Female participants, white participants, and those from higher socioeconomic backgrounds were over-represented in TeenCovidLife (Huggins et al., 2022). The study also had a relatively small sample size. Although thousands of pupils participated in at least one of the three TeenCovidLife surveys, complete data across all three surveys was only available for 255 pupils, and of these only 56 were boys. In addition, the question used to measure bullying referred to bullying “in school”, and was not presented to participants at Time 1. As such we were unable to establish whether pupils experienced bullying outside of school or prior to Time 2.

Despite its limitations, the present research has a number of implications for how adolescent mental health can be understood in light of the Covid-19 pandemic. Our results found that lockdown was not the most stressful time for the pupils in this study, nor was it associated with lower wellbeing than when pupils were in school. In contrast, experience of being bullied was clearly associated with higher perceived stress and lower wellbeing, especially when pupils were attending school. This suggests that, when it comes to pupils’ wellbeing, policy makers and practitioners should focus less on the impact of lockdown and more on the impact that the pandemic is continuing to have on young people. Making school a less stressful environment should be an additional priority (Soneson et al., 2022). For example, decision makers at all levels should prioritise clarity and timeliness when making decisions about stressful school milestones, such as exams.

Both victims and perpetrators of bullying show low levels of belief-in-others (e.g. lower levels of perceived peer support, school support and family coherence; Arslan et al., 2021). This suggests that interventions to improve relationships in any of these areas of life could ameliorate the impact of bullying. One such intervention is a teacher-run peer support programme, in which older pupils are trained to help younger pupils learn skills in connectedness, sense of self, and school citizenship (Ellis, Marsh & Craven, 2009). Teachers facilitating these programmes could prioritise pupils who show more indicators of potential victimisation, such as emotional and behavioural problems or reductions in school performance (Arslan et al., 2021; Vaillancourt et al., 2021). Work by Arslan and colleagues (2021) found that positive psychological orientations (e.g. high levels of self-efficacy, empathy, self-awareness) may be protective against the aforementioned harmful effects of bullying. They suggest that school-based mental health practitioners could therefore focus on strengths-based interventions such as Acceptance and Commitment Therapy (Hayes et al., 2006). Beyond interventions focused on specific pupils, school practitioners could also move towards more positive educational approaches by drawing on resources such as the SEARCH framework (Waters & Loton, 2019). This framework seeks to help schools embed positive psychology approaches to education which include: Strengths, Emotional management, Attention and awareness, Relationships, Coping, and Habits and goals (Waters & Loton, 2019).

In line with findings from Soneson et al. (2022), it is apparent that Covid-19 lockdowns had different effects on different individuals. Indeed, the present findings contribute to tentative evidence that pupils who experienced the most difficulties before the pandemic (such as those who were bullied or had high levels of depression symptoms) may have been most likely to experience an improvement in wellbeing after the pandemic began (Soneson et al., 2022; Houghton et al., 2022). Practitioners should be aware that the same disruptive event (such as a lockdown) could have opposing effects on the mental health of different groups of pupils, and that these groups may require different kinds of support. Going forward, it is essential that research seeks to identify which aspects of the Covid-19 lockdown were associated with positive outcomes, for whom, and how these aspects could be used to improve the school environment (Soneson et al., 2022).

In summary, the present research found that during lockdown pupils who had experienced bullying showed similar levels of perceived stress to those who had not been bullied. However, once the pupils returned to school, perceived stress was markedly higher in those who had been bullied than those who were not bullied. This pattern did not emerge for wellbeing scores; instead, pupils who experienced bullying showed consistently lower wellbeing scores across time. These findings highlight that pupils’ levels of perceived stress during the first months of the Covid-19 pandemic varied according to their experiences of bullying. This contributes to a growing body of work which suggests that the impact of the Covid-19 pandemic on adolescent mental health varies according to social context and the pre-existing circumstances of each young person.
